# Reactive Extrusion Printing for Simultaneous Crystallization‐Deposition of Metal–Organic Framework Films

**DOI:** 10.1002/anie.202117240

**Published:** 2022-02-19

**Authors:** Fatimah Al‐Ghazzawi, Luke Conte, Christopher Richardson, Pawel Wagner

**Affiliations:** ^1^ Intelligent Polymer Research Institute and ARC Centre of Excellence for Electromaterials Science AIIM Faculty Innovation Campus University of Wollongong North Wollongong NSW 2522 Australia; ^2^ Al-Nasiriyah Technical Institute Southern Technical University Thi-Qar Iraq; ^3^ School of Chemistry and Molecular Bioscience Faculty of Science Medicine and Health University of Wollongong North Wollongong NSW 2522 Australia

**Keywords:** HKUST-1, Metal–Organic Frameworks, Reactive Extrusion Printing, Thin Films

## Abstract

Reactive extrusion printing (REP) is demonstrated as an approach to simultaneously crystallize and deposit films of the metal–organic framework (MOF) Cu_3_btc_2_ (btc=1,3,5‐benzenetricarboxylate), also known as HKUST‐1. The technique co‐delivers inks of the copper(II) acetate and H_3_btc starting materials directly on‐surface and on‐location for rapid nucleation into films at room temperature. The films were analyzed using PXRD, profilometry, SEM and thermal analysis techniques and confirmed high‐quality Cu_3_btc_2_ films are produced in low‐dispersity interconnected nanoparticulate form. The porosity was examined using gas adsorption which showed REP gives Cu_3_btc_2_ films with open interconnected pore structures, demonstrating the method bestows features that traditional synthesis does not. REP is a technique that opens the field to time‐efficient large‐scale fabrication of MOF interfaces and should find use in a wide variety of coating application settings.

## Graphical abstract

Scalable fabrication methods for metal–organic framework (MOF) films are crucial for integrating this class of materials with applications. Reactive extrusion printing (REP) co‐delivers reactive inks that rapidly form micron‐thick interconnected nanoparticulate films of Cu_3_btc_2_ (HKUST‐1) on‐demand and on‐location.

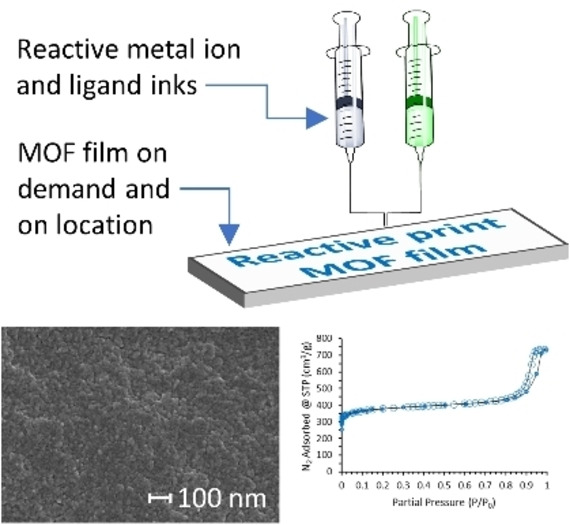

Metal–organic frameworks are a class of crystalline porous solids constructed from organic linkers that bridge metal ion nodes into reticulated lattices. Their precisely‐defined pore structures coupled with their modular construction endows unrivalled structural tuneability and the potential to be tailored as solid‐phase adsorbents for gases,^[1]^ water purification,^[2]^ energy recovery surfaces,^[3]^ pharmaceutical separations,^[4]^ chemical sensing^[5]^ and as heterogeneous catalysts.^[6]^ MOFs, as crystalline materials, are usually produced in powdered forms that require shaping and structurization processes for application in practical settings. The formation of films is of great interest for integration with device technologies for microelectronics, microfluidics and sensing technologies^[7]^ but this structural form also needs consideration on larger scales in the production of catalytic coatings, for example. Creating uniform large area MOF films is particularly challenging and has rarely been achieved. Scalable methods include step‐by‐step spraying^[8]^ and hot‐pressing.^[9]^ Direct deposition of MOF films on the substrate and at the desired location holds many advantages and therefore methods for simultaneously controlling positioning and deposition at micro and macroscales are critical for advancing the use and applicability of this class of material.

We thought liquid ink printing technologies, which are used widely industrially, present opportunities for controlled MOF film growth. We targeted reactive extrusion printing (REP) for co‐delivery of separate solutions of metal and ligand inks to the substrate surface for rapid nucleation and film formation (Figure 1). We aimed to demonstrate REP as a straightforward technique with potential to be deployed at scale for coverage of macroscale areas with MOF films without additional fabrication steps encountered with lithographic methods or the need for binders or surfactants or other additives. Extrusion printing using pre‐formed MOF particles^[10]^ in gels is known^[11]^ but to the best of our knowledge reactive extrusion printing of MOFs and MOF films represents a new advance. We recently reported reactive inkjet printing for the spatial postsynthetic modification of pre‐formed MOF films.^[12]^ Extrusion differs from inkjet printing in that droplets are forced physically from the nozzle, usually under mechanical force.

**Figure 1:**
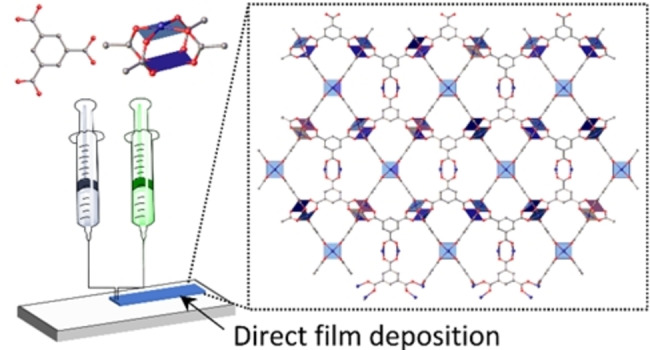
A schematic of reactive extrusion printing. The molecular structures of H_3_btc and a copper(II) acetate paddle wheel unit are shown atop the reactive inks along with an inset view of the crystal structure of Cu_3_btc_2_.

Herein, we demonstrate the technique of REP for producing micron‐thick interconnected nanoparticulate films of a well‐studied prototype model MOF, Cu_3_btc_2_, also known as HKUST‐1. The structure of Cu_3_btc_2_ is reticulated through square dicopper paddlewheel nodes and the trigonal btc bridging linkers into an open lattice with pore diameters of 5, 11 and 13 Å (Figure 1) and films of this MOF have been prepared on glass,^[13]^ gold,^[14]^ copper^[15]^ and copper oxide^[16]^ and alumina surfaces^[17]^ and by many solution‐based methods including *in situ* crystallization,^[18]^ secondary growth,^[19]^ dip‐coating,^[20]^ and electrochemical deposition.^[21]^ Many of these methods present practical difficulties to implement at scale because they require a concomitant increase in reactor size. We note that inkjet printing was used to fabricate patterned Cu_3_btc_2_ films on paper, plastic, and textile substrates from a single stable ink that required oven drying to initiate nucleation for each layer by removal of high boiling point solvents (DMSO and ethylene glycol) and then immersion and washing in methanol solution after each print.^[22]^

We pursued experiments by preparing inks in solvents in which Cu_3_btc_2_ is commonly synthesized (DMF, ethanol and water). The first ink system was 1–1 vol./vol. DMF–ethanol for the copper(II) ion ink and the H_3_btc ink, respectively, and the second system was water for the copper(II) ion ink and 96 % ethanol for the H_3_btc ink. Inks were prepared from 0.05 M to 0.30 M to study the effect of concentration in the REP process. The highest concentration of H_3_btc in ethanol was 0.15 M as we found this was close to saturation at room temperature without leading to problems of precipitation. Copper(II) acetate was chosen as the copper ion source to promote rapid MOF formation.^[23]^

It is well‐known that the properties of the inks in combination with the properties of the substrate are responsible for adhesion and printing resolution. We measured surface tensions of inks with and without the highest concentration of copper(II) acetate used in the study (0.30 M) by the pendant droplet method at the liquid–air interface using a goniometer (Figure S1) and the results are summarized in Table S1.^[24]^ We found, as expected, all tensions decreased upon metal ion incorporation which leads to better surface wettability.

As a proof of concept, we chose to work with glass slide substrates for their ease of functionalization and low cost. The glass surfaces were treated by O_2_ plasma and by self‐assembled monolayer (SAM) formation using aminopropyltriethoxysilane (APTES)^[25]^ to enhance binding of copper ions and H_3_btc molecules by surface‐exposed hydroxyl groups from plasma treatment or by terminal amino groups on the APTES SAM.

The effects of the surface treatments were gauged from water contact angles on the native and O_2_ plasma‐treated glass surfaces for 3, 6, 9 and 12 minutes and the SAM functionalized glass (Figure S2). Longer plasma treatment times gave decreasing water contact angles (from 54° for the native glass surface to virtually 0° for the surface treated for 12 minutes) and increasing surface energies (Table S2), indicating increasing substrate surface hydrophilicity, in agreement with previous reports.[^26^, ^27^, ^28^] We anticipated the wide spreading of the ink droplets at plasma treatment times longer than 6 minutes would lead to unacceptable printing resolution. Therefore, contact angles were also measured for the solvent systems on the O_2_ plasma treated glass for 6 minutes and the APTES SAM and these showed the expected decrease (Figure S3; Table S3). The APTES SAM showed the least hydrophilic properties suggesting it may be the surface offering the best control over printing linewidth resolution.

Printing was performed using a Sheline 1820 printer with computer‐controlled stage and printer head, and an extrusion deposition system (Figure S4A). The substrate was moved at a speed of 193 mm min^−1^ in X‐ and Y‐directions and the distance between the nozzles and the substrate (Z‐direction) was adjusted to 0.50 mm for all prints. A bespoke nozzle holder was 3D‐printed to accommodate two stainless steel needles (22 gauge, internal diameter 0.41 mm) that were connected to 1 mL syringes containing the copper(II) acetate and H_3_btc inks (Figure S4B). The needle tips were positioned to touch, allowing rapid mixing of extruded inks. Ink extrusion from the syringes was controlled by a syringe pump at 6.0 μm s^−1^ giving a flow rate of 0.10 mL s^−1^ (Supporting Information). An area of a glass slide of size 79×28 mm is printed in approximately four minutes. A movie of printing is provided in the Supporting Information (Video S1). Crystallization‐deposition takes place within seconds on the surface and the printed areas appear dry after removal from the printer.

A first set of experiments assessed the effect of plasma treatment time as we thought the wide spreading of droplets at treatment times longer than 6 minutes would lead to unacceptable printing resolution for each solvent system. Fast nucleation and crystallization kinetics were desired in all experiments and therefore copper(II) acetate was used in a two‐fold excess of H_3_btc.^[23]^ This was implemented by using copper(II) acetate inks at twice the concentration of H_3_btc inks and at the same flow rate. This resulted in consumption of H_3_btc, which was observed in preliminary experiments with lower metal to ligand ratios, and facilitated easy removal of the excess copper(II) acetate from the printed areas by washing with ethanol. Printing was performed at the highest ink concentrations for this set of experiments (0.30 M copper(II) acetate and 0.15 M H_3_btc) as we hypothesized higher concentrations would give the fastest nucleation and best conditions for film formation. Optical micrographs from REP for each solvent system (Figure S5) confirmed that print quality indeed decreased significantly when the glass surface was plasma‐treated longer than after 6 minutes and indicated that the better results were obtained for the ethanol–water ink system. We also note that water has been shown to have good properties as a growth modulator for growing Cu_3_btc_2_ MOF films.[^20a^, ^20b^]

Experiments to determine the effect of ink concentration on film formation were carried out as, in principle, film thickness should be controllable by ink concentration and the number of printed layers. The results presented in Figure S5 show that acceptable continuous films with reasonably uniform coverage only formed in the water–ethanol ink system at 0.30 M copper(II) acetate to 0.15 M H_3_btc. The results from the DMF–ethanol ink system led to unacceptable coverage and consistency. Close optical inspection revealed important and consistent differences in the films over repeated experiments. Prints from the DMF–ethanol solvent system were less uniform with wider spreading and cracked into plate‐like fragments during drying (Figure 2A). Films from water–ethanol, however, were far superior and without any apparent mechanical defects (Figure 2B–D). This would be attributable to better properties of the water–ethanol ink combination (lower boiling point, higher contact angles, and higher surface tension)[^27^, ^28^, ^29^] and therefore this ink combination was used for all further studies.

**Figure 2:**
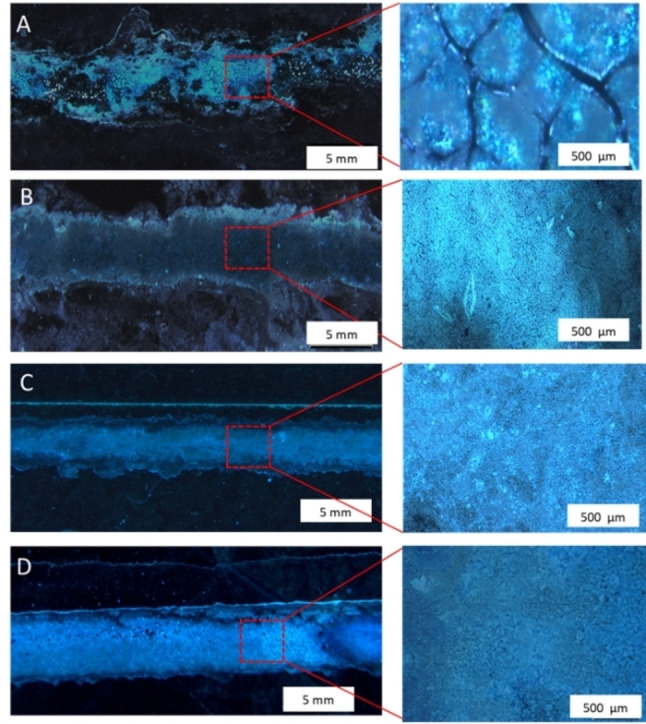
Optical microscopy images of printed Cu_3_btc_2_ lines using 0.30 M copper(II) acetate and 0.15 M H_3_btc inks. A) 1–1 DMF–ethanol ink on plasma treated glass with four print repetitions, B) H_2_O–ethanol ink on plasma treated glass with four print repetitions, C) H_2_O–ethanol ink on APTES treated glass with a single print, and D) H_2_O–ethanol ink on APTES treated glass with two print repetitions.

On the plasma‐treated glass surface, the first layer created was a not a uniform and continuous film. The second layer improves the coverage, and a uniform and continuous line is formed by the fourth print. In contrast, the APTES‐modified surface gives a uniform and continuous line in the first print.

We then determined the additivity in film thickness with the number of printed layers. Figure 3 displays the results from profilometry after one, two and four print repetitions using the water–ethanol system at 0.30 M copper(II) acetate and 0.15 M H_3_btc on plasma‐treated and APTES‐modified glass surfaces. Profilometry over sections of the films showed that although the surfaces were relatively rough, film thickness increases near‐linearly (≈1.5–2 μm) with the number of printed layers, and this was consistent over repeated experiments.

**Figure 3:**
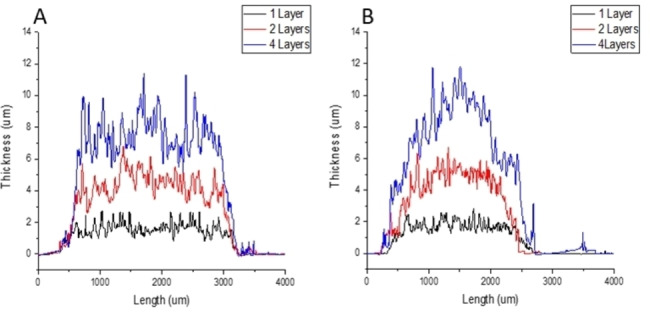
Thickness profile of Cu_3_btc_2_ films. A) On plasma‐treated glass, and B) on APTES‐treated glass. Layer one shown in black, layer two in red and layer four in blue.

It was clearly observed through this set of experiments that the APTES‐modified surface resulted in a better resolution of printed lines with less variation (1.46±0.03 mm to 2.44±0.23 mm from one to four layers) than the plasma‐treated surface (2.33±0.16 mm to 3.55±0.11 mm from one to four layers). This can be correlated to the less hydrophilic surface properties of the APTES SAM surface (Table S3). We also suggest that the amino terminal groups of the SAM are more efficient at binding interactions with copper ions and H_3_btc than the hydroxyl groups on plasma‐treated glass surfaces. Interestingly, although the linewidth is smaller on the APTES‐functionalized surface, the line thickness is not very different for the first print layer compared to the plasma‐treated glass surface, and repeat prints lead to a less even coverage and thickness of the lines on the APTES SAM; a typical result obtained from profilometry is shown in Figure 3.

The crystallinity and phase purity of the films after four print repetitions on plasma‐ and APTES‐treated surfaces were examined direct from the printed surfaces (areas ≈1.5×1.5 cm) using PXRD. Figure 4 shows the diffractograms for the samples and a sample of Cu_3_btc_2_ synthesized via a room‐temperature solvothermal method,^[30]^ as a comparison. The patterns are essentially identical, indicating that the films are pure Cu_3_btc_2_ and with no discernible orientation effects.^[31]^ Thermal analysis using TGA–DSC measurements also showed identical characteristics between samples prepared by REP and bulk powders prepared by the traditional solvothermal method (Figure S6).

**Figure 4:**
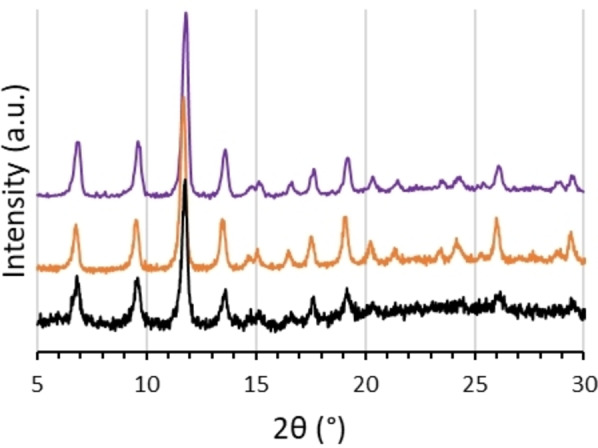
Diffractograms for printed Cu_3_btc_2_ on plasma‐ and APTES‐treated surfaces (black and orange traces, respectively) and Cu_3_btc_2_ prepared solvothermally (purple).

Analysis using SEM revealed the surface of each film has randomly oriented interconnected nanocrystals with a similar shape and that sometimes there were larger crystallites on the film surface (Figure 5). Particle size analysis using ImageJ software showed that REP produces small crystals with good dispersity with average sizes of 36±9 nm for the plasma‐treated surface and 28±5 nm for the APTES modified surface. We also analysed the film surface from printing using the DMF–ethanol ink and found this gives considerably larger crystals with an average size of 88±27 nm (Figure 5C). The distributions are shown in Figure S7. We suggest this outcome might relate to a longer time for crystal growth due to the slower evaporation of the higher boiling point solvent. These results identify that changing the solvent system and therefore the physicochemical properties of the ink is potentially a method to tune crystal size in the printed films.

**Figure 5:**
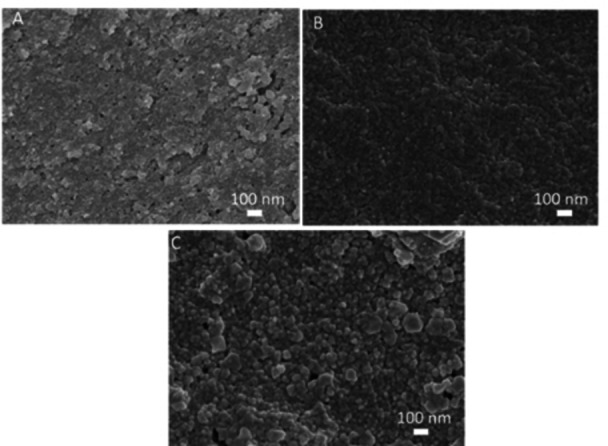
SEM images of Cu_3_btc_2_ prepared from A) H_2_O–ethanol ink on plasma‐treated glass, B) H_2_O–ethanol ink on APTES SAM, and C) DMF–ethanol ink on plasma‐treated glass.

The textural properties of the films were examined by N_2_ gas adsorption at 77 K after removal from the surfaces and activation under dynamic vacuum at 393 K for 16 hours. Figure 6 shows the adsorption–desorption isotherms obtained display type IV behaviour with a H1 hysteresis loop. The hysteresis was explored further by scanning isotherms (Figure S8) and revealed an open interconnected pore system in the printed samples, indicating Cu_3_btc_2_ synthesized by REP has additional porosity.^[32]^ The Brunauer–Emmett–Teller surface area was 1473 m^2^ g^−1^, which is typical of defect‐free Cu_3_btc_2_. The defect density was assessed via X‐ray photoelectron spectroscopy,^[33]^ which corroborated the defect‐free nature of the particles (Figure S9–S11).

**Figure 6:**
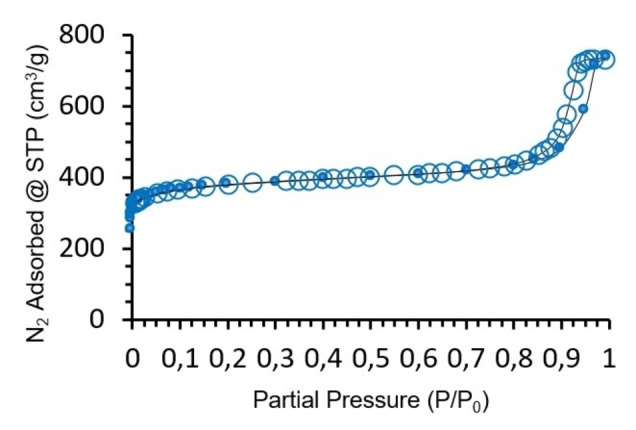
N_2_ adsorption–desorption isotherms for reactive‐printed Cu_3_btc_2_ at 77 K. Adsorption points are shown as filled circles and desorption points as unfilled circles. A line for adsorption and desorption is provided as a guide for the eye.

In conclusion, we have developed reactive extrusion printing (REP) as a new, rapid and straightforward technology for preparing micron‐thick macroscale MOF films. REP utilises co‐delivery of miscible ink solutions of metal and ligand components from different nozzles directly to the surface for on‐location film formation. In contrast to the previously reported depositions, this technique avoids the use of preformed particles or the need to develop formulations for stable single inks and the method can produce surface patterns. The films produced by this technique were uniform, of high quality, and free of mechanical defects. Our study indicated that a key factor for creating quality films is substrate surface preparation. Bulk‐scale diffraction, thermal and surface area techniques confirmed the structure, purity, and porosity of the films. SEM analysis showed the film surfaces were composed of connected intergrown Cu_3_btc_2_ nanocrystals and that some size control was gained using different solvent inks. Our results suggest ink systems that vary considerably from traditional MOF solvent systems for crystal growth may be used and the method may be best suited to MOFs with fast nucleation kinetics. We envision REP can be applied to many MOFs, coordination polymers, and nanomaterials systems.

## Conflict of interest

The authors declare no conflict of interest.

## Supporting information

As a service to our authors and readers, this journal provides supporting information supplied by the authors. Such materials are peer reviewed and may be re‐organized for online delivery, but are not copy‐edited or typeset. Technical support issues arising from supporting information (other than missing files) should be addressed to the authors.

Supporting Information

Supporting Information

## Data Availability

The data that support the findings of this study are available in the supplementary material of this article.
