# Supplementary material for: Reactive Extrusion Printing for Simultaneous Crystallization‐Deposition of Metal–Organic Framework Films
Source: Angew Chem Int Ed Engl. 2022 Feb 19;61(15):e202117240. doi: 10.1002/anie.202117240 (PMC9303373; doi:10.1002/anie.202117240)
Supplement: Supplementary file 2 — Supporting Information [file ANIE-61-0-s001.pdf]

Angewandte Chemie
Eine Zeitschrift der Gesellschaft Deutscher Chemiker

Supporting Information

Reactive Extrusion Printing for Simultaneous Crystallization-Deposition of Metal–Organic Framework Films

F. Al-Ghazzawi, L. Conte, C. Richardson, P. Wagner**

Author Contributions

F.A.-G. Data curation:Lead; Investigation:Lead; Writing – review & editing:Supporting
L.C. Data curation:Supporting; Investigation:Supporting; Writing – review & editing:Supporting
C.R. Conceptualization:Lead; Data curation:Supporting; Formal analysis:Lead; Investigation:Supporting; Supervision:Equal; Writing – original draft:Lead; Writing – review & editing:Lead
P.W. Formal analysis:Supporting; Supervision:Equal; Writing – review & editing:Supporting

Supporting Information

Table of Contents

Materials and Methods 2
Surface tension and contact angle measurements 4
Thermogravimetric analysis-differential scanning calorimetry, TGA-DSC 9
SEM Particle size analysis 10
N_2 Isotherms & BET surface area calculations 11
XPS Analysis 12
References 16

Materials and Methods

Trimesic acid (H_3btc) was purchased and used as received from Sigma-Aldrich and copper(II) acetate monohydrate ($Cu(OAc)_2 \cdot H_2O$)was purchased and used as received from Fluka.

Extrusion printing was carried out using a Sheline 1820 Machinery printer. The stage and printer head system, and the extrusion deposition system, were controlled by Link CNC-EMC software. The stage and the printer head were movable in X Y and Z direction. The substrate was moved at a speed of 193 mm/min in the longitudinal and lateral directions (X Y motion). The gap between the nozzles and the substrate (Z motion) was adjusted manually to 0.5 mm and constant for all printing experiments.

Disposable syringes (1.00 mL) were used as ink reservoirs. Stainless steel needles were used as nozzles (22 GE). One nozzle extruded the copper salt ink and the other extruded the H_3BTC solution at the same time. The flow speeds of the reactive inks were controlled at speed 6 µm/s and the volume of ink flow rate from the syringes (1.00 mL) was 0.104 mL/s. After reactive printing, the formed film was washed twice with EtOH and twice with acetone and then dried at room temperature, unless stated otherwise.

Glass microscope slides (76.2 × 25.4 mm) were used as substrates. O_2 Plasma treatment was done using a Harrick plasma system PDC002for 3, 6, 9, or 12 minutes with a chamber pressure set at 1000 milliTorr. A CAST3 USA KINO goniometer was used to measure contact angles and surface tensions. Values were obtained from measurements of five different drops for each surface and ink. The sessile drop method was used for measuring contact angle, and the pendant drop method was used for measuring surface tension at room temperature. Ink droplets were dispensed on to the substrate by the printer and software program for recording and analysing contact angle and surface tension.

Optical micrographs were recorded using a Leica M205A or a Leica DM6000 each fitted with CCD cameras. The Leica M205A was used at scales from 1-10 mm and the Leica DM6000 from 500-25 µm. A JEOL JSM-7500 was used to study morphologies of all the samples at 15 keV.

A GBC Multi Materials Analyser X-ray diffractometer was used to record patterns of printed MOF films. Data were recorded in the 2θ range of (5-30°) with a step size of 0.05 at $1°min^{-1}$. Patterns were recorded directly on the surface they were printed on.

TGA-DSC was performed using NETZSCH STA 449F3 Jupiter simultaneous analyser, connected to a multi-channel temperature controller (TR 004). Typically, ~8 mg of the sample was heated from 25 to 1000 ˚C at 10 ˚C per min under N_2/ O_2 flow (20 cm^3 min^{-1}). The data analysis was recorded and carried out on the NETZSCH Proteus version 6.1.0 software system.

Gas adsorption studies were carried out at the Wollongong Isotope and Geochronology Laboratory using a Quantachrome Autosorb MP instrument and high purity nitrogen (99.999 %) gas. Surface areas were determined using Brunauer-Emmett-Teller (BET) calculations.

The image-J64-bit software program was used to measure the distribution and the particles size of SEM images. The SEM image was imported then processed by re-setting the scale of the software based on the scale of SEM image. Then 100 particles were selected randomly, and their diameters were measured manually.

XPS was carried out by Dr Bin Gong and Dr Songyan Yin at the Surface Analysis Laboratory, Mark Wainwright Analytical Centre, UNSW using an ESCALAB250Xi instrument from Thermo Scientific, UK with a background vacuum better than 2×10^{-9} mbar and mono-chromated Al Kα (energy 1486.68 eV) X-ray source (120 W, 13.8 kV x 8.7 mA) on spot sizes of 500 micrometres. The pass energy was 100 eV for survey scans and 20 eV for region scans. Further details are provided with the spectra.

Ligand ink preparation: Trimesic acid (10.50 mg, 0.05 mmol) or (21.00 mg, 0.10 mmol) or (31.50 mg, 0.15 mmol) were dissolved in 1-1 DMF-ethanol (1.00 mL) for the first ink system and in ethanol (1.00 mL) for the second ink system in glass vials to obtain concentrations of 0.05 M, 0.10 M, and 0.15 M, respectively.

Metal ink preparation: Copper(II) acetate monohydrate (20.00 mg, 0.10 mmol) or (40.00 mg, 0.20 mmol) or (60.00 mg, 0.30 mmol) were dissolved in 1-1 DMF-ethanol (1.00 mL) for the first ink system and H_2O (1.00 mL) for the second ink system in glass vials to obtain inks with concentrations 0.10 M, 0.20 M, and 0.30 M, respectively.

APTES SAM formation procedure: Glass slides were treated by O_2 plasma for 10 minutes then immediately placed into a toluene solution of 2% (vol./vol.) 3-aminopropyltriethoxysilane for 24 hours under an atmosphere of argon at room temperature. Afterwards, the glass slide was washed three times with toluene under nitrogen. Finally, the slide was rinsed three times with acetone and ethanol and then dried.

Surface tension and contact angle measurements

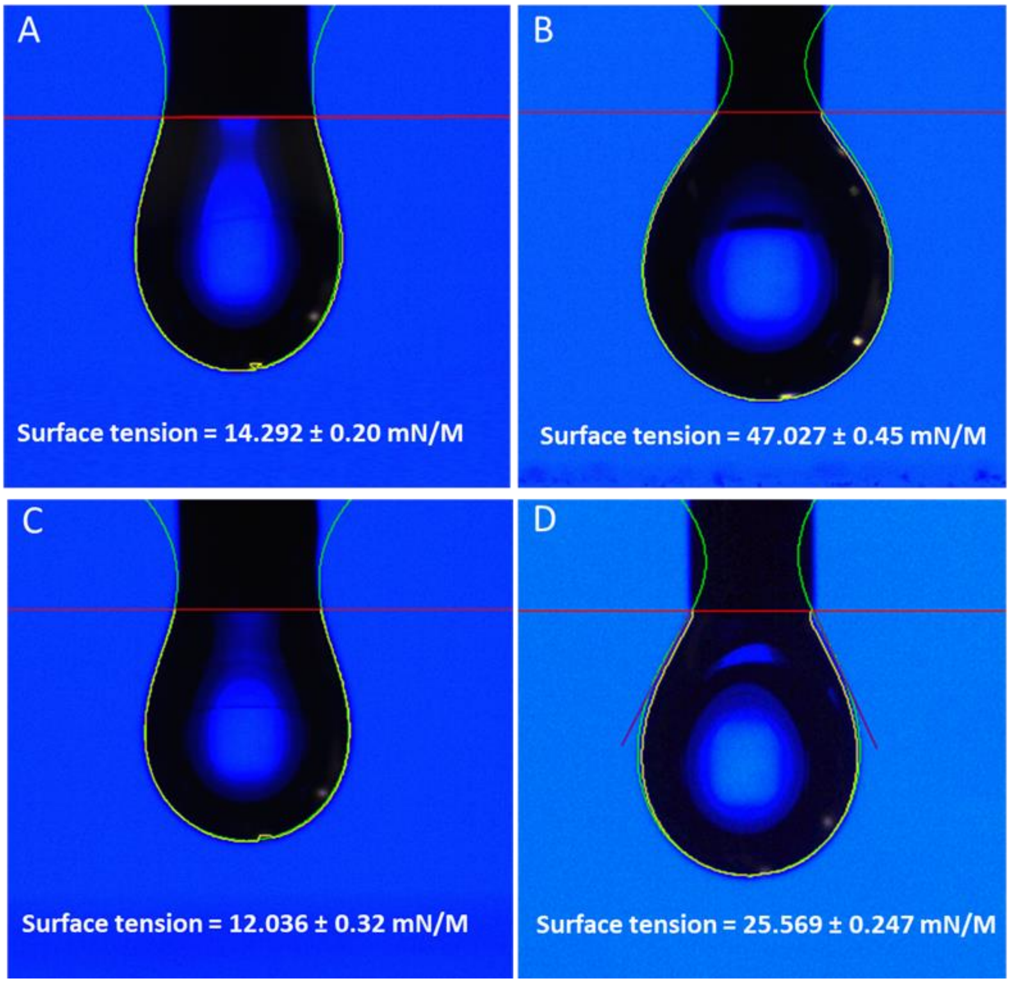

Figure S1 Surface tension measurements of 0.30 M copper(II) acetate inks. A) 1-1 DMF-ethanol, B) H_2O, C) Ethanol and D) 1-1 H_2O-ethanol.

Table S1 Surface tensions of ink solutions at room temperature.

Solvent system	Copper(II) acetate ink concentration (M)	Surface tension (mN/M)	Surface tension pure liquid (mN/M)
1-1 DMF-ethanol	0.30	14.29 ± 0.20	36
H_2O	0.30	47.03 ± 0.45	72
Ethanol	0.30	12.04 ± 0.32	22
1-1 H_2O-ethanol	0.30	25.57± 0.25	37

*Standard deviations were obtained from measurements of five different drops.

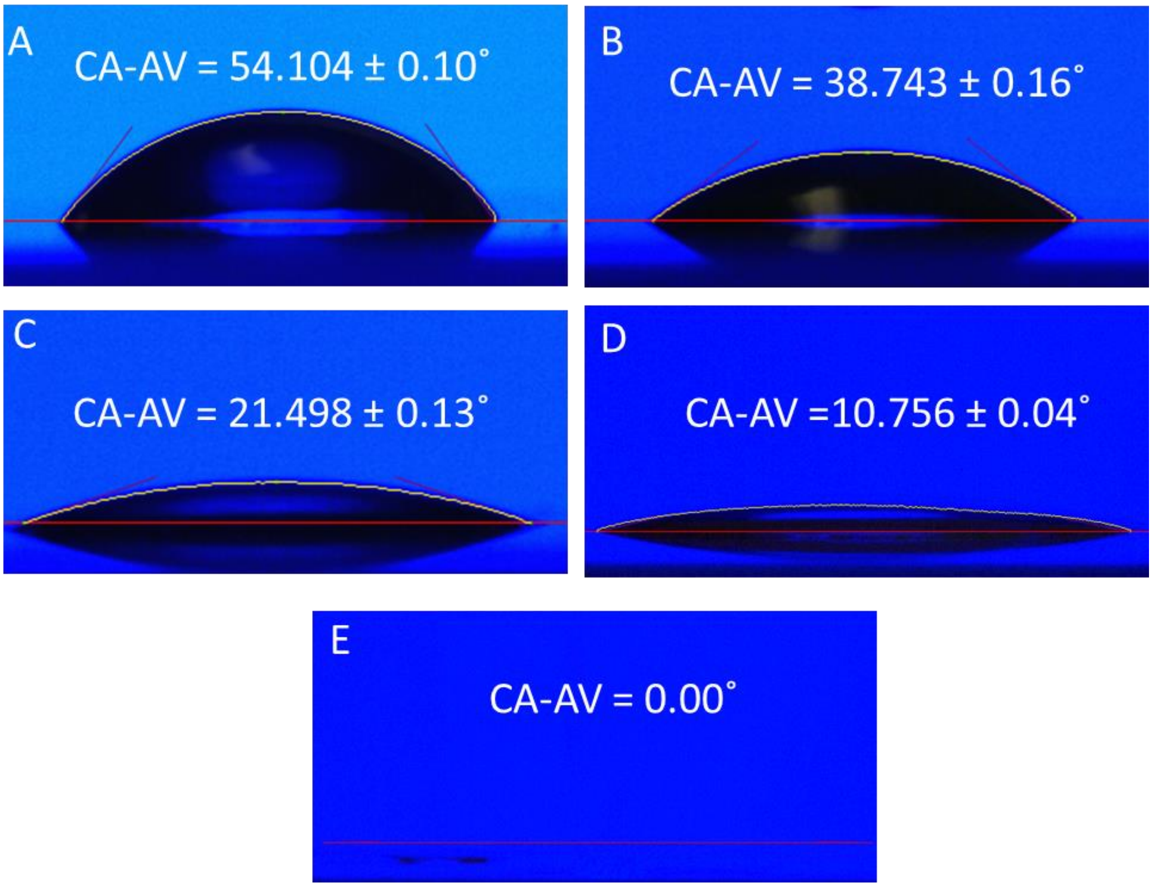

Figure S2 Water contact angles on A) untreated glass substrate and glass substrates treated with O_2 plasma for B) 3 min, C) 6 min, D) 9 min, and E) 12 min.

Table S2 A summary of contact angles and surface energies of water droplets on the glass as a function of O_2 plasma treatment time.

O_2 plasma-treatment time (min)	Contact angle (°)	Surface energy (mN/m)
Untreated	54.10 ± 0.21*	50.07 ± 0.10
3	38.74 ± 0.51	58.89 ± 0.16
6	21.49 ± 0.13	66.82 ± 0.08
9	10.76 ± 0.04	70.07 ± 0.03
12	0.00 ± 0.03	71.20 ± 0.01

*Standard deviations were obtained from measurements of five different drops.

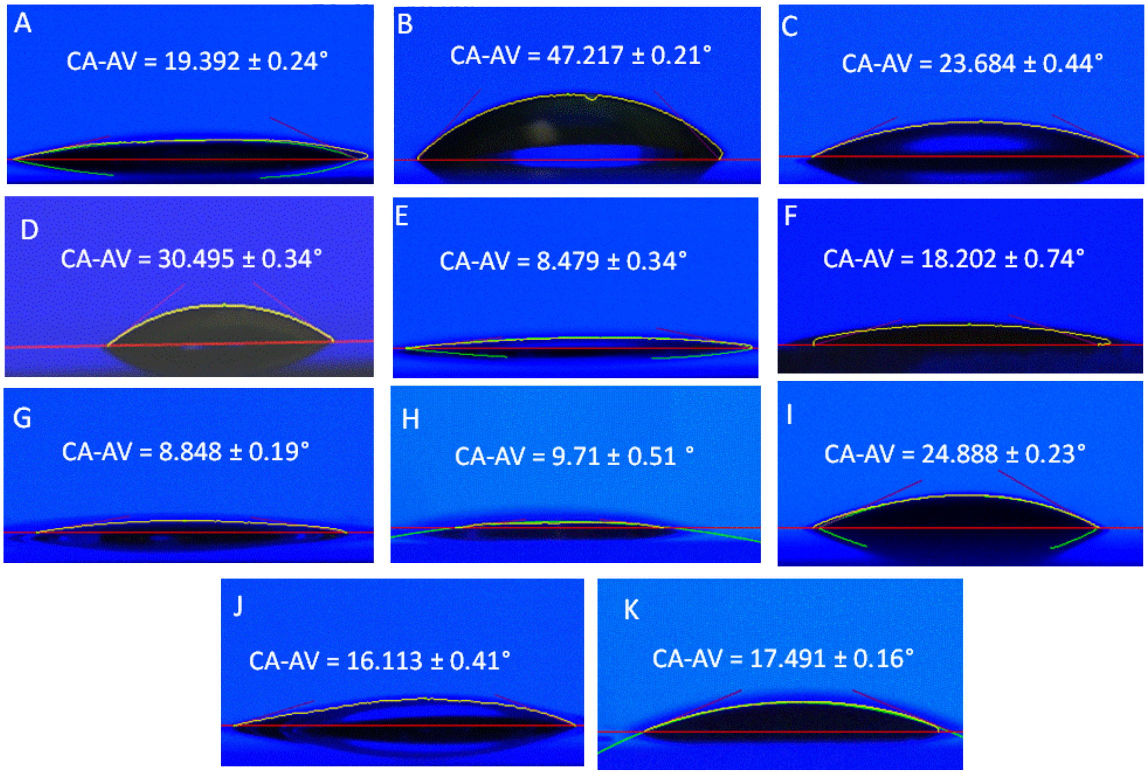

Figure S3 Surface contact angles of inks on substrate before surface treatment: A) 1-1 DMF-ethanol, B) H_2O, C) EtOH, and D) 1-1 H_2O-ethanol. Surface contact angle of inks after treatment by O_2 plasma for 6 minutes: E) 1-1 DMF-ethanol, F) H_2O, G) EtOH; and H) 1-1 H_2O-ethanol. Surface contact angle of inks after APTES SAM formation: I) H_2O, J) EtOH; and F) 1-1 H_2O-ethanol.

Table S3 A summary of contact angles and surface energies measured for solvent systems on O_2 plasma treatment surfaces at 0.30 M copper(II) acetate ink concentration.

Ink system	Contact angle before plasma treatment (°)	Contact angle after plasma treatment (°)	Surface energy (mN/m)	
			Before	After
1-1 DMF-ethanol	19.39 ± 0.24	8.48 ± 0.34	67.56 ± 0.14	70.48 ± 0.17
H_2O	47.22 ± 0.21	18.20 ± 0.74	53.99 ± 0.15	67.93 ± 0.10
Ethanol	23.68 ± 0.44	8.85 ± 0.19	65.87 ± 0.18	70.33 ± 0.13
1-1 H_2O-ethanol	30.49 ± 0.25	9.71 ± 0.51	62.79 ± 0.19	70.21 ± 0.18

*Standard deviations were obtained from measurements of five different drops.

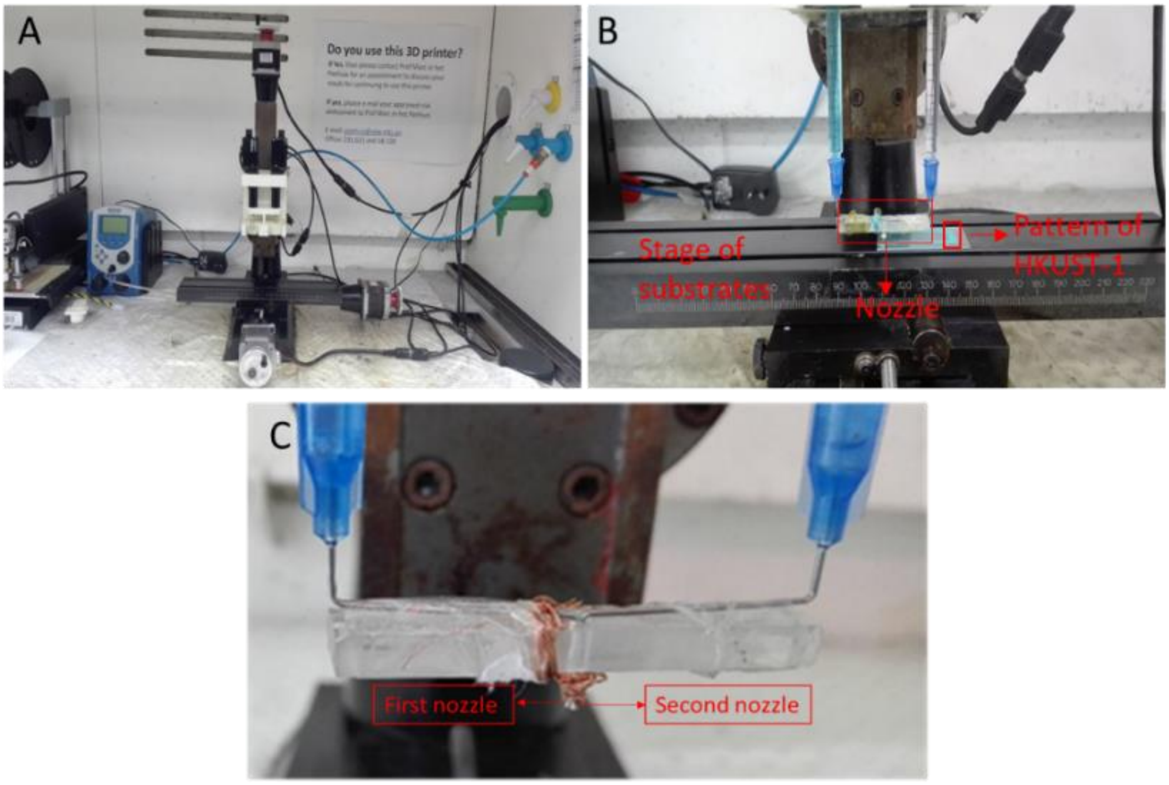

Figure S4 A) Photo of the extrusion printer, B) Close-up view of the syringes and substrate stage and C) Close up view of the nozzles.

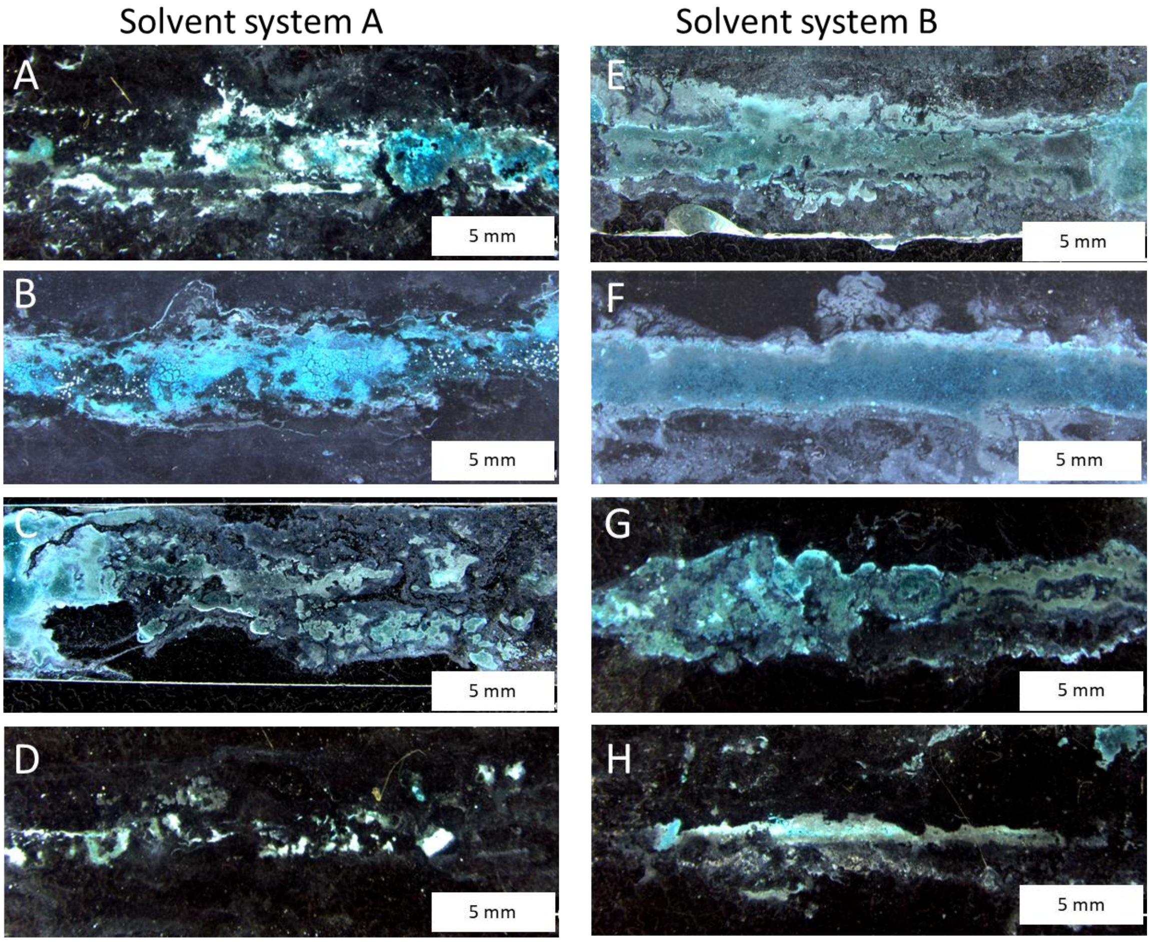

Figure S5 Effect of O_2 plasma treatment time on the quality and resolution of Cu_3btc_2 printed patterns. Results for the DMF-ethanol solvent system (solvent system A) are shown in A)-D); and the results for H_2O-ethanol (solvent system B) are shown in E)-H). A) and E) on untreated glass, B) and F) at 3 min treatment time, C) and G) at 6 min treatment time, and D) and H) at 9 min treatment time. Scale bars = 5 mm.

Thermogravimetric analysis-differential scanning calorimetry, TGA-DSC

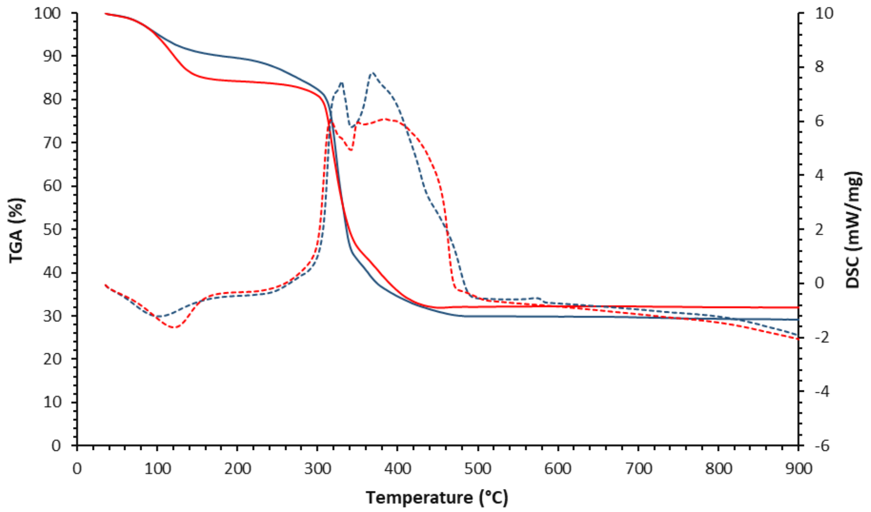

Figure S6 TGA-DSC curves of reactive-printed Cu_3btc_2 (red) and solvothermally-produced[1] Cu_3btc_2 (blue). TG (solid lines) and DSC (dashed lines).

SEM Particle size analysis

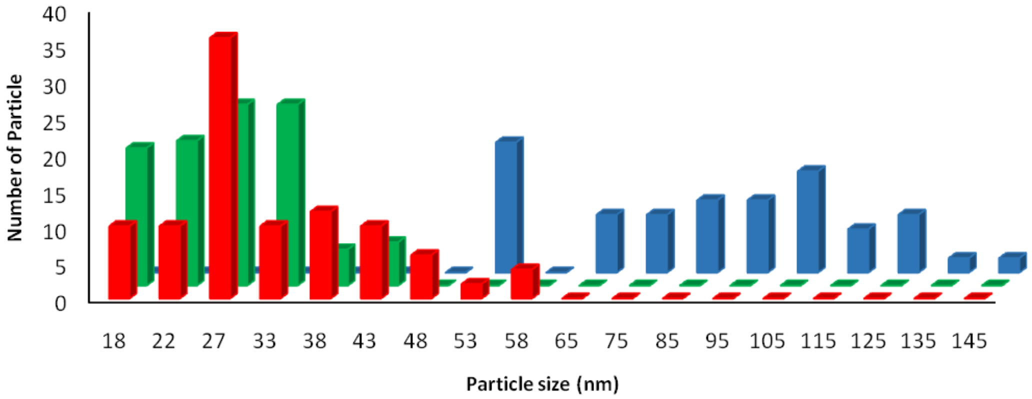

Figure S7 Particle size distribution of Cu_3btc_2 printed from 1-1 H_2O-ethanol ink on plasma-treated glass surface for 6 minutes (red bars), from 1-1 H_2O-ethanol ink on the APTES-modified surface (green bars) and from 1-1 DMF-ethanol plasma-treated glass surface for 6 minutes (blue bars).

N_2 Isotherms & BET surface area calculations

Table S4 BET Summary for Cu_3btc_2 produced by reactive extrusion printing.

Slope =		2.364
Intercept =		5.59E-04
Correlation coefficient, r =		1.0000
C constant =		4229.425
Surface Area =		1473.099 m²/g
Relative Pressure P/Po	Volume @ STP (cc/g)	1 / [W((Po/P) - 1)]
8.00831E-03	331.9543	1.9458E-02
9.01819E-03	333.0897	2.1860E-02
9.97608E-03	334.1178	2.4130E-02
1.20603E-02	335.7478	2.9091E-02
1.50532E-02	338.0448	3.6174E-02
2.87278E-02	345.7477	6.8447E-02
4.09247E-02	350.9553	9.7282E-02

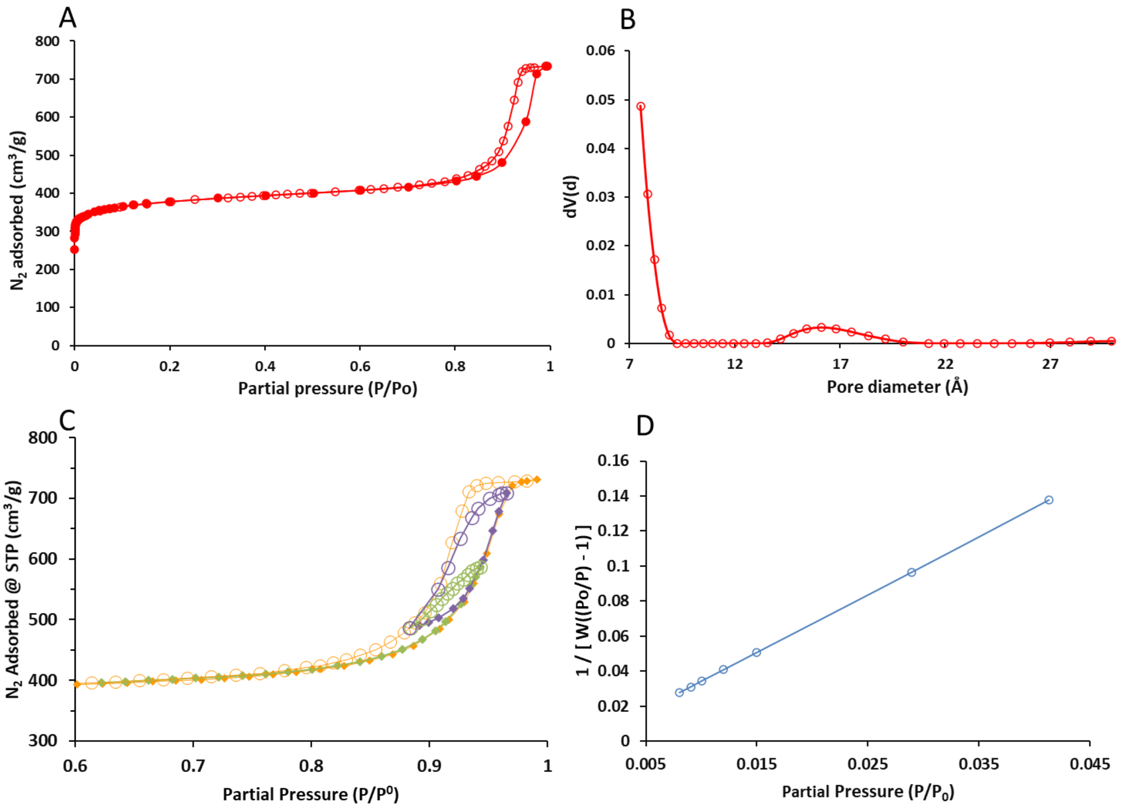

Figure S8 A) N_2 adsorption-desorption isotherm at 77 K; B) pore diameter distributions of printed HKUST-1; C) Hysteresis scanning isotherms of printed Cu_3btc_2, cycle one (orange), cycle two (green), cycle three (purple); and D) BET plot. Open symbols represent adsorption and closed symbols represent desorption on A) and C).

XPS Analysis

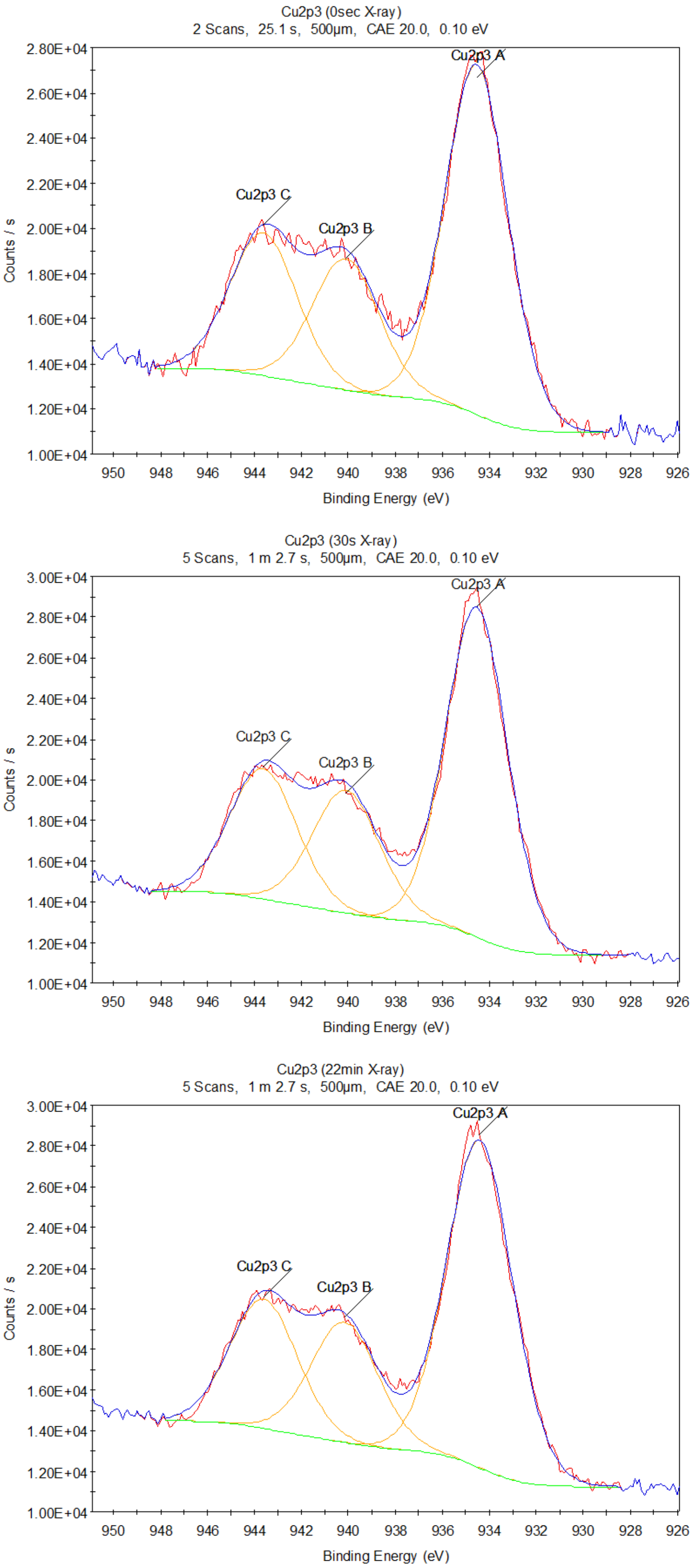

Figure S9 $Cu2p^{3/2}$ XPS analysis under various X-ray exposure times of Cu_3btc_2 produced by reactive extrusion printing.

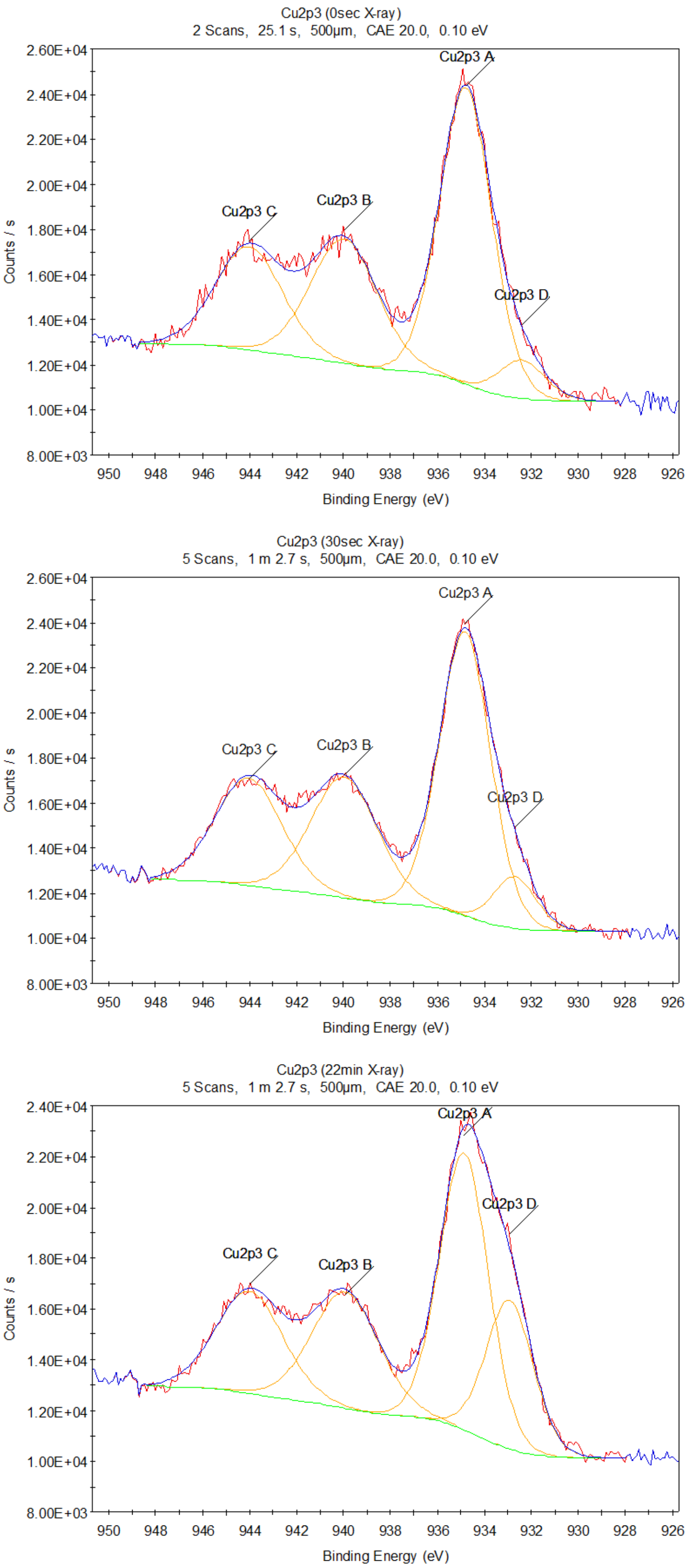

Figure S10 Cu2p$^{3/2}$ XPS analysis under various X-ray exposure times of Cu_3btc_2 produced by solvothermal reaction.[1]

Table S5 XPS Peak summary for Cu_3btc_2 produced by solvothermal reaction.

Peak Table :	0 sec X-ray exposure				
	Name	Peak BE	FWHM eV	Area (P) CPS.eV	Atomic %
Cu(II)	Cu2p3 **A***	934.78	2.61	37148.18	47.43
Cu(II) sat 1	Cu2p3 **B**	939.97	3.37	20113.82	25.74
Cu(II) sat 2	Cu2p3 **C**	944.01	3.37	16734.71	21.46
Cu(I)	Cu2p3 **D**	932.39	2.29	4218.82	5.38
**Peak Table :**	30 sec X-ray exposure				
	Name	Peak KE	FWHM eV	Area (P) CPS.eV	Atomic %
Cu(II)	Cu2p3 **A**	551.86	2.56	34834.27	45.2
Cu(II) sat 1	Cu2p3 **B**	546.72	3.37	19510.4	25.38
Cu(II) sat 2	Cu2p3 **C**	542.64	3.37	17359.31	22.62
Cu(I)	Cu2p3 **D**	553.98	2.11	5241.69	6.8
**Peak Table :**	22 min X-ray exposure				
	Name	Peak BE	FWHM eV	Area (P) CPS.eV	Atomic %
Cu(II)	Cu2p3 **A**	934.86	2.45	29018.5	38.42
Cu(II) sat 1	Cu2p3 **B**	939.92	3.37	16693.85	22.15
Cu(II) sat 2	Cu2p3 **C**	943.99	3.37	14705.56	19.55
Cu(I)	Cu2p3 **D**	932.92	2.37	15023.41	19.87

* Labels A-D refer to the peaks listed in Figure S10.

XPS Discussion:

We compared samples from our reactive extrusion printing to a literature method. We found extensive reduction of copper(II) to copper(I) using Al Kα radiation (1486.6 eV) at 70 W with 50 eV pass energy with ~20 scans. Reducing the pass energy to 20 eV (same step size of 0.1 eV) and reducing the number of scans gave good results and this is the data presented. The samples produced by reactive extrusion printing showed no presence of copper(I), which has been used as a marker of defects.[2] This has to be used with caution as we demonstrate above that increasing X-ray exposure time increases the proportion of copper(II) photoreduction for Cu_3btc_2 samples with defects, even with low dose X-rays.

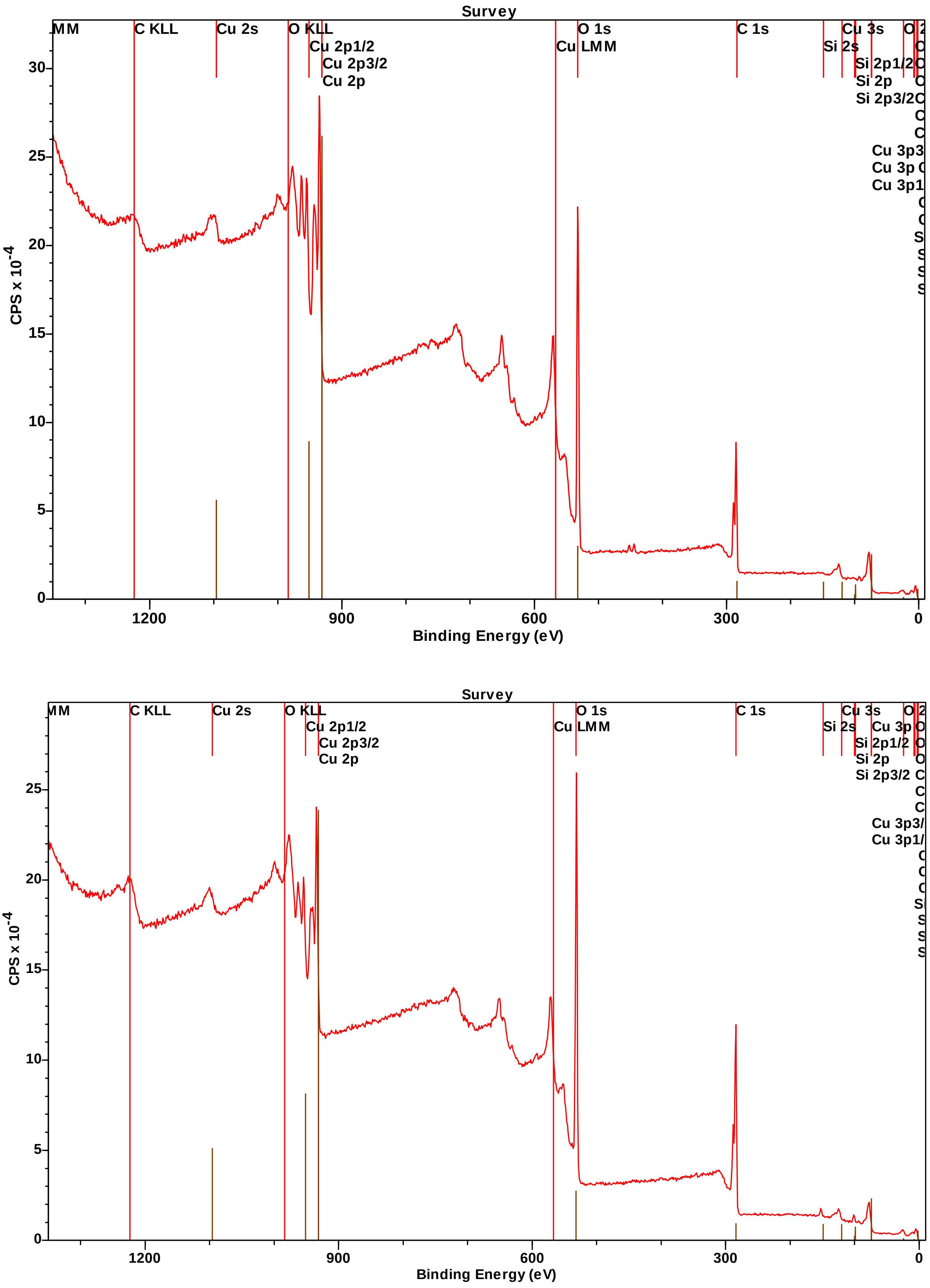

Figure S11 Survey XPS spectra of Cu_3btc_2 produced by reactive extrusion printing (top) and solvothermal reaction[1] (bottom). Both show the presence of trace silicon from glassware in which the samples were printed on and stored in.

References

[1] Z.-Q. Li, L.-G. Qiu, T. Xu, Y. Wu, W. Wang, Z.-Y. Wu, X. Jiang, *Mater. Lett.* **2009**, *63*, 78-80.
[2] K. Müller, K. Fink, L. Schöttner, M. Koenig, L. Heinke, C. Wöll, *ACS Appl. Mater. Interfaces* **2017**, *9*, 37463−37467
